# Preferred product attributes of a multipurpose vaginal ring: Findings from a phase 1 trial

**DOI:** 10.3389/frph.2023.1148134

**Published:** 2023-03-30

**Authors:** Elizabeth E. Tolley, Homaira Hanif, Andrea Thurman, Vivian Brache, Gustavo F. Doncel

**Affiliations:** ^1^ FHI 360, Global Health and Population Research, Durham, NC, United States; ^2^ CONRAD and Eastern Virginia Medical School, Norfolk, VA, United States; ^3^ Profamilia, Santo Domingo, Dominican Republic

**Keywords:** HIV prevention, contraception, vaginal ring, product attributes, acceptability, preferences

## Abstract

**Introduction:**

Most women face multiple and co-occurring risks from unwanted pregnancy, human immunodeficiency virus (HIV) and other sexually transmitted infections (STIs) at some point during their lifetime. While a range of contraceptive methods exist and options for HIV prevention are increasing, to date, only male and female condoms provide multipurpose protection from both pregnancy and disease.

**Methods:**

From September 2017 to December 2018, 60 women from the United States and the Dominican Republic, randomized 1:1 to continuous or interrupted use and 4:1 to active vs. placebo ring, participated in a Phase I trial to assess the safety and tolerability of a three-month multipurpose intravaginal ring (IVR) containing the antiviral tenofovir and the contraceptive levonorgestrel. This study examines survey responses from all participants and qualitative data from a subset of 17 women to assess acceptability of and preferences for IVR characteristics.

**Results:**

Overall, women liked the concept of a multipurpose IVR and found it easy to insert and remove. Initial concerns about the size or thickness of the ring generally disappeared with use experience. Women weighed trade-offs between the ease of continuous use for a longer duration against concerns about hygiene and discoloration of the ring when left in place during menses. Whether randomized to continuous or interrupted use, most women found ring attributes (size, thickness, flexibility) very acceptable. They provided recommendations *via* survey and qualitative interviews for ring modifications that would further increase acceptability. Insights into women's use experiences also suggest the need for clear counseling messages and introduction strategies that can facilitate women's choice and use of prevention methods.

**Discussion:**

Study findings suggest that a multipurpose IVR would make a valuable contribution to women's sexual and reproductive health options, and that both continuous and interrupted use strategies may be preferred.

## Introduction

Across their lifespan, most women face multiple and co-occurring risks from unwanted pregnancy, HIV and other STIs (1). Between 2015 and 2019, the global burden of unintended pregnancies averaged approximately 121 million per year; more than half (61%) ended in abortion (2, 3). In 2021, approximately 1.5 million people were newly diagnosed with HIV; approximately half of new infections globally were in women, but almost two-thirds of infections in sub-Saharan Africa (63%) were among women and girls (4).

Although a range of contraceptive products exist and options for HIV prevention are increasing, women often face barriers to uptake and use of single indication prevention products, let alone use of several products to meet multiple needs. Whether for contraception or HIV prevention, barriers include women's perceptions of side effects, ease or burden of product use requirements, partner disapproval or ability to use discreetly, cost and access issues, and broader sociocultural norms (5, 6). The development of multipurpose prevention technologies (MPTs) could improve women's sexual and reproductive health (7). However, these products must be acceptable and easy to adhere to, if they are going to address women's multiple health needs (8, 9).

Intravaginal rings (IVR) are a promising platform to deliver multiple agents. To date, they have been used to deliver steroids for contraception or postmenopausal therapy, and anti-retroviral agents for HIV prevention (10). In addition, an MPT IVR containing the antiviral tenofovir and the contraceptive levonorgestrel is currently under development. Two randomized, placebo-controlled Phase I trials, conducted among low-risk women in the United States (US) and Dominican Republic (DR), evaluated the safety and tolerability of a 90-day intravaginal ring, used either continuously, for 15–90 days, or over three interrupted cycles of 28 days (11, 12). Both regimens were found to be safe and well tolerated (12). Furthermore, acceptability of the ring, whether used continuously or in an interrupted fashion, was high (13). In comparison to other prevention products, whether for pregnancy or HIV prevention, most women preferred a product that delivered two-in-one protection. While about half of participants reported changes to their menstrual cycle after initiating product use, the most common change was a reduction in bleeding quantity or duration, a change that most women liked (13).

The study, like past studies of vaginal rings also provided some insights into how vaginal ring attributes affect acceptability, including perceptions related to ring size and color (14, 15), the ease or difficulty of placement and removal (16), and concerns about whether the ring might move around in the body or be felt during sex (17, 18). In this paper, we build on the previously published acceptability data (13) to provide a more in-depth examination of user preferences for modifiable product attributes of a multipurpose IVR containing tenofovir and levonorgestrel. As new sexual and reproductive health products move through their critical path from discovery to introduction, the need to obtain timely feedback from the product's potential end users has become increasingly apparent (19).

## Methods

A Phase I randomized, placebo-controlled trial was conducted between September 2017 and December 2018 to evaluate the safety and tolerability of a MPT IVR containing the antiviral tenofovir (TFV) and the contraceptive levonorgestrel (LNG) (11). A total of 60 women from two sites (Norfolk, Virginia and Santo Domingo, Dominican Republic) who were at low risk for both pregnancy and HIV were randomized 1:1 to continuous or interrupted use of a 90-day IVR, with a 4:1 ratio of receiving an active or placebo ring. A secondary objective of this trial was to assess women's experiences using the TFV/LNG IVR, including preferences for IVR attributes related to ring dimensions and continuous vs. intermittent use patterns. These acceptability data were collected through two strategies. All participants were administered survey questions at three timepoints (baseline, within the first month of use, and at three months just after ring removal). In addition, a subset of a maximum of 10 participants per site were invited to take part in qualitative interviews at months one (M1) and three (M3). These interviews were conducted in person and in Spanish in the Dominican Republic, and *via* mobile phone in English in the U.S. Interviewers in both sites were trained in qualitative data collection.

In this paper, we examine survey responses at the three-month follow-up visit (M3) on acceptability of vaginal ring characteristics (e.g., size, thickness, flexibility) with response options based on a six-point scale from very unacceptable to very acceptable. We also examine whether specific changes in ring characteristics would make the ring more, or less, acceptable. These data are disaggregated by regimen and site. The data collection instruments and approach to analysis for the acceptability objective of the trial have been described previously (13). Briefly, bivariate analyses (Fisher's exact tests and Chi-squared tests) were conducted to determine statistically significant differences. In addition, we followed a thematic analysis process to analyze and present textual data from the subset of qualitative interviews related to women's perceptions of ring characteristics.

This study was approved by the Chesapeake IRB (now Advarra; Pro00022358) at Eastern Virginia Medical School and the Institutional Review Board of Profamilia (IORG0001979) and National Bioethics Council (Conabios IORG003206). All participants provided written informed consent to participate in the clinical trial. Participants of qualitative interviews were purposively selected by an unblinded study statistician to represent the continuous and interrupted regimens. They provided a separate written informed consent that included permission to be audio-recorded.

## Findings

Reported previously (13), a total of 47 women completed baseline surveys and 18 women, 11 from the DR and 7 from the US, participated in the qualitative sub-study. In both sites, participants' mean age was 37, although women in the DR were more likely to be living with a partner (84%) than women in the US (50%). About one-third of women from the DR (32%) had ever experienced using an IVR, compared to 45% of US participants. Women in the US were more likely to use vaginal hygiene products, compared to women in the DR (Table 1).

**Table 1:** Menstrual hygiene management, by regimen, agent, and site.

	Regimen		Agent		Site		
	Continuous	Interrupted	Active	Placebo	DR	US	Total
Product normally used during menses (%)	*N* = 25	*N* = 22	*N* = 37	*N* = 10	*N* = 25	*N* = 22	*N* = 47
Probability for Fisher's exact test	*p* = 0.355		*p* = 0.344		*p* < 0.001		
Menstrual pads	76	63.6	70.3	70	96.0*	40.9*	70.2
Tampons	0.0	9.1	5.4	0.0	0.0*	9.1*	4.3
Both pads and tampons	24.0	22.7	24.3	20	4.0*	45.5*	23.4
Other	0.0	4.6	0.0	10	0.0*	4.6*	2.1

The star (*) denotes a probability value <0.05 using a Fisher's exact test.

Overall, women in this trial liked the idea of a multipurpose product and found the MPT vaginal ring acceptable. In the M3 survey, most participants reported IVR attributes to be very acceptable with flexibility (87.5%), mode of insertion and removal (82.5%), smoothness (77.5%) and color (70%) ranking highest. Participants expressed somewhat lower levels of acceptability towards changes in color over time, although less than 10% found these changes to be even “a little” unacceptable, and none found them to be “somewhat” or “very” unacceptable. There were no significant differences between acceptability of ring characteristics by regimen, agent (active vs. placebo) or site (Table 2).

**Table 2:** Acceptability of ring characteristics by regimen and site at M3.

	Regimen		Site		
	Continuous	Interrupted	DR	US	Total
	(*n* = 21)	(*n* = 19)	(*n* = 24)	(*n* = 16)	(*n* = 40)
**Size (%)**					
Very unacceptable	0.0	0.0	0.0	0.0	0.0
Somewhat unacceptable	4.8	0.0	0.0	6.3	2.5
A little unacceptable	4.8	0.0	4.2	0.0	2.5
A little acceptable	14.3	5.3	16.7	0.0	10.0
Somewhat acceptable	19.1	36.8	20.8	37.5	27.5
Very acceptable	57.1	57.9	58.3	56.3	57.5
**Thickness (%)**					
Very unacceptable	0.0	0.0	0.0	0.0	0.0
Somewhat unacceptable	9.5	0.0	0.0	12.5	5.0
A little unacceptable	4.8	0.0	4.2	0.0	2.5
A little acceptable	9.5	15.8	20.8	0.0	12.5
Somewhat acceptable	14.3	21.1	20.8	12.5	17.5
Very acceptable	61.9	63.2	54.2	75.0	62.5
**Flexibility (%)**					
Very unacceptable	0.0	0.0	0.0	0.0	0.0
Somewhat unacceptable	0.0	0.0	0.0	0.0	0.0
A little unacceptable	4.8	0.0	0.0	6.3	2.5
A little acceptable	4.8	5.3	8.3	0.0	5.0
Somewhat acceptable	4.8	5.3	4.2	6.3	5.0
Very acceptable	85.7	89.5	87.5	87.5	87.5
**Color (%)**					
Very unacceptable	0.0	0.0	0.0	0.0	0.0
Somewhat unacceptable	0.0	0.0	0.0	0.0	0.0
A little unacceptable	0.0	5.3	0.0	6.3	2.5
A little acceptable	9.5	10.5	16.7	0.0	10.0
Somewhat acceptable	14.3	21.1	12.5	25.0	17.5
Very acceptable	76.2	63.2	70.8	68.8	70.0
**Smoothness (%)**					
Very unacceptable	0.0	0.0	0.0	0.0	0.0
Somewhat unacceptable	0.0	0.0	0.0	0.0	0.0
A little unacceptable	0.0	5.3	0.0	6.3	2.5
A little acceptable	4.8	10.5	8.3	6.3	2.5
Somewhat acceptable	9.5	15.8	12.5	12.5	12.5
Very acceptable	85.7	68.4	79.2	75.0	77.5
**Way it is inserted/removed (%)**					
Very unacceptable	0.0	0.0	0.0	0.0	0.0
Somewhat unacceptable	4.8	0.0	0.0	6.3	2.5
A little unacceptable	0.0	5.3	0.0	6.3	2.5
A little acceptable	0.0	0.0	0.0	0.0	0.0
Somewhat acceptable	19.1	5.3	12.5	12.5	12.5
Very acceptable	76.2	89.5	87.5	75.0	82.5
**Change in color over time (%)**					
Very unacceptable	0.0	0.0	0.0	0.0	0.0
Somewhat unacceptable	4.8	0.0	4.2	0.0	2.5
A little unacceptable	0.0	10.5	0.0	12.5	5.0
A little acceptable	19.1	10.5	16.7	12.5	15.0
Somewhat acceptable	23.8	21.1	29.2	12.5	22.5
Very acceptable	52.4	57.9	50.0	62.5	55.0

Qualitative interviews provide more insight into IVR attributes including women's perspectives on the ease or difficulty of insertion and removal, the acceptability of the ring's size, thickness, smoothness, and flexibility, as well as color and experiences during use, such as side effects and comfort during sex.

### Insertion and removal

During the initial ring insertion visit, women were offered the opportunity to practice insertion and removal. Most participants found insertion and removal easy. For example, a 36-year-old US participant equated the insertion process to “inserting a regular tampon.” She went on to explain, *“They’re easy to use, just fold and insert so you don’t need to be a rocket scientist to figure it out.”* (Continuous use, #207) In a similar way, a 34-year-old participant from the DR explained, *“Because you only have to grab the ring and take it out. You enter your finger, and you find it. And when you touch it, you try to pull it, slowly. Yes, it was easy.”* (Continuous user, #123) Six women, some from each site, had used an IVR previously, either NuvaRing as a contraceptive, or in a different clinical trial. Some of these women equated their experience with the study product to those previous experiences.

*A* few women (*n* = 3) expressed initial concern about inserting the IVR properly. In such cases, the staff were able to provide guidance. One U.S. participant doubted her ability to correctly insert the ring, explaining that she was “*not completely comfortable with that, only because when I did put it in myself, it wasn’t far back enough. So, I would prefer to be with them (clinic staff) when I put it back in.”* (Continuous use, #212) However, with practice, even women who expressed initial reservations about insertion or removal described the process as *easy* or *smooth sailing*.

*Wow that experience was, wow! I inserted it very well, because I had already inserted rings before […] But to take it out, wow … . We took over 20 min for me to be able to take it out. …  She (clinician) then had me lie down, and she said “Look, I am going to try to help a little to show you how you are going to do it. But imagine that it's not me, but you, that is going to do it.” And then she did it, and she asked me to insert it again, and … everything was perfect. So now I know how to remove it. Now I have inserted it twice and removed it twice. (39-year-old mother of 3 in the DR, #124)*

### Size and thickness

Relatedly, more than half of sub-study participants (*n* = 11)—all but two of them from the DR, were initially concerned about the size or thickness of the ring. At first sight, women worried about whether it would fit inside their bodies, whether the ring might move around during daily activities, or whether their partner might feel the ring during sex. Those concerns usually disappeared after insertion or initial use. When asked what her first thoughts were, a DR participant exclaimed,

*A little big! I had never inserted anything in there, so …  I mean, not even my fingers, I don't. …  Yes. To insert something up there, no, no, never. In my vagina, no. I saw that ring and I said wow, and I touched it. It was a little thick. But what I saw is not the same as what I feel. I was surprised by what I saw, but after I inserted it, everything is perfect. It doesn't bother me or anything. (43-year-old mother of 4 in the DR, #121)*

### Smoothness and flexibility

Most women found the smoothness and flexibility of the ring to be *fine*. As one DR participant described, *it's plastic or rubber …  is not uncomfortable.* Another said, *the color, the flexibility that you can bend it easily… it is all good.* Only two women, both from the DR, initially described the ring as “*rough”*.

*When I Saw it? I thought, “Oh my God, that is thick and rough!” [Laughter.] I thought it was going to be smaller! I thought it'd be something. Oh my God. I found it to be rough. Oh my God. But what can you do? Onward. But it was, it was easy. (32-year-old mother of two in DR, #119)*

### The use experience

Few women (*n* = 4) reporting experiencing any side effects from ring use and none worried about symptoms they experienced. Three women described some changes to their menstrual cycles, accompanied by headache or nausea, that were noticeable. The fourth described stronger mood swings after using the ring. However, none reported these changes as problems, and most wondered whether they changes were due the ring itself, or to the biopsies or their normal menstrual cycle.

*There's something I mentioned to my coordinator. I think it might've been a headache or—I can't remember what the symptom was but I think she had said it was more likely related to my biopsy. (INT 2) For the most part, positive. Towards the end I did notice just one side effect. I noticed that I would get pretty bitchy just before my period. That was something that I haven't really experienced in the years of having my period. (27-year-old mother of one in US on active cyclic ring, #215)*

Regardless of any initial concerns about the size, thickness or flexibility of the ring, most qualitative sub-study participants (*n* = 11) reported not being able to feel the ring during their day-to-day activities, including during sex. Indeed, when asked about their sexual experience during ring use, most women described disliking the requirement to use condoms during the trial. While a few women (*n* = 4) reported that their partners were able to feel the IVR during sex, only two women reported this to be a problem. A 31-year-old participant from the US site explained,

*The only other negative thing I remember from it was that during sex, my husband told me that he could feel it and that it was almost scratching him. After I talked to the doctor and study coordinator about it, they thought that it was probably that hard piece that doesn't bend and that maybe that was rubbing up against him or something … . Then, depending on which time we were having sex, sometimes it didn't bother me and other times I would feel it. I would feel like I wasn't necessarily feeling the ring, but it was just feeling like a pain in my lower abdomen. It would feel like something was kind of hitting up against your side. That was uncomfortable. I remember those two things during sex that were sporadic. Sometimes it was fine for him and sometimes it wasn't. Sometimes it was fine for me and sometimes it wasn't. (31-year-old U.S. woman, no children on continuous ring, #212)*

In contrast, another participant whose partner could feel the ring remarked,

*We have great sex, it's awesome. What you're probably asking is if he felt the ring and he did. Sometimes when we have sex, he can like feel the ring, but it doesn't bother him. He can kind of just like feel that it's there. (28-year-old US woman, one child on continuous ring, #217)*

### Color

When asked about any changes in the ring color over time, women remarked on two different aspects. Women generally liked the “*transparency”* of the ring and several noted that the ring “*appears to have a white medication”* inside. A Dominican woman in the interrupted use arm further described how “*When it was removed the second time in the second month it was changing. The liquid was going away. The third time it was completely gone.” (26-year-old DR woman, mother of 3, #117)* For some, the ability to see the medication inside was a benefit, “*it allows you to see if anything is going wrong. If it changes colors, then you know (that the medicine is leaving the ring)*.” (*39-year-old mother of 3 in the DR* #124).

In addition, several participants also described a change in the exterior appearance of the ring over time. Women generally stated that such changes were due to menstruation and were therefore acceptable. Only two women, both from the DR, found changes to ring color after menses less than appealing.

*A little ring, a white little ring. Then, when the menstruation comes the color changes. As the bleeding came, it changed color, it was like brown now. Completely brown …  AND WHAT DO YOU THINK ABOUT THE COLOR CHANGE? It was because of the medication or the menstruation. Do you understand me? It gets dirty, that's what I think. (26-year-old DR woman with 3 children, interrupted use, #117)*

### Duration

Women generally preferred a ring that could be used continuously for three months or, as on DR participant said, “*Yes, for my whole life! Put it in and that's it. That it just stays right there. I didn’t have any issue with the time.”* Several women compared using a longer-acting IVR to using an IUD. However, when considering continuous use for three or more months, women raised several caveats related to menstrual hygiene management. First was the idea that they should be able to remove the ring periodically to clean it. A US participant explained,

*So now cleanliness is something I’ve thought about. You know, if this is something that goes out on the market and it is a three-month ring, if women take it out quickly to rinse it off, is that okay? That's something I'm sure other women are going to wonder about. (45-year-old US woman with 7 children, interrupted use, 214)*

A second concern for several participants in the US, but none in the DR, related to the compatibility of continuous IVR use with use of menstrual hygiene products, including tampons and the menstrual cup.

*I guess one sort of concern I have is, for the purpose of the study, I was told I cannot use my menstrual cup, but I can use tampons. I feel that had a bit to do with why I chose to do the study because I don't necessarily know if I would have if I had to only use pads. I'm curious if it's a thing for the purposes of the study or if using a cup with this product would be a complication. That would affect my interest in it if it were a product on the shelf. (27-year-old US woman, one child, interrupted use, 215)*

### Recommendations for IVR modifications

At M3, participants rated whether potential changes to the MPT IVR would make the ring less or more acceptable (Table 3). At least half of participants indicated that making the ring smaller would increase acceptability. Overall, about 40% of participants recommended making the ring thinner and/or more flexible, while smaller proportions of participants recommended changes to color or smoothness. Interestingly, women in the interrupted use regimen were significantly more likely to recommend providing an applicator for insertion or removal (42.9%) compared to women in the continuous use arm (10.5%).

**Table 3:** Acceptability of potential changes to ring characteristics by regimen and site at M3.

	Regimen		Site		
	Continuous	Interrupted	DR	US	Total
	(*n* = 21)	(*n* = 19)	(*n* = 24)	(*n* = 16)	(*n* = 40)
**More Acceptable (%)**					
Make ring size smaller	57.1	42.1	50.0	50.0	50.0
Make ring thinner	42.9	36.8	45.8	31.3	40.0
Increase flexibility of ring	33.3	42.1	37.5	37.5	37.5
Provide applicator to insert ring	42.9*	10.5*	25.0	31.3	27.5
Make the color opaque	28.6	21.1	25.0	25.0	25.0
Make the ring less slippery	28.6	10.5	25.0	12.5	20.0
Make ring stiffer	0.0	5.3	0.0	6.3	2.5
Make ring size bigger	0.0	0.0	0.0	0.0	0.0
Make ring thicker	0.0	0.0	0.0	0.0	0.0
**Less Acceptable (%)**					
Make ring size bigger	52.4	57.9	50.0	62.5	55.0
Make ring thicker	57.1	42.1	54.2	43.8	50.0
Make ring stiffer	42.9	57.9	50.0	50.0	50.0
Make ring size smaller	9.5	15.8	12.5	12.5	12.5
Make ring thinner	9.5	15.8	12.5	12.5	12.5
Provide applicator to insert ring	0.0*	26.3*	12.5	12.5	12.5
Increase flexibility of ring	4.8	15.8	12.5	6.3	10.0
Make the color opaque	0.0	15.8	4.2	12.5	7.5
Make the ring less slippery	0.0	15.8	0.0	18.8	7.5

The star (*) denotes a probability value < 0.05 using a χ^2^ test of association.

Data from the qualitative sub-study followed a similar pattern. About half of IDI participants found the ring acceptable just as it was. “*No, the size is good. The color, the flexibility that you can bend it easily… it is all good. For me, everything. I wouldn’t change anything.”* (37-year-old DR woman in the interrupted use arm, #118) A few others suggested changes not for themselves, but because others might prefer such modifications. For example, when recommended that the ring be *thinner*, she added, “*But, even though I didn’t find it to be difficult, maybe someone would find it uncomfortable. And that would make it easier.”* (36-year-old DR woman, continuous use, #125).

## Discussion

Participants in early-stage prevention clinical trials may differ from the end-users who eventually use the products being evaluated. Nevertheless, the value of engaging potential end-users earlier in the product development pipeline has been increasingly acknowledged (9, 20, 21). In this trial, participants were likely to be at lower risk for pregnancy, HIV and other STIs. They were also willing to be randomized to an experimental product or a placebo, come for frequent clinic visits, undergo biopsies, abstain from sex and/or use condoms. Over a third of participants had some experience using an IVR, either in previous research or as a contraceptive method. Yet, they provided important insights into attributes of the 90-day TFV/LNG IVR and potential strategies to support their introduction and use in the future.

A first insight is that concerns about size, thickness, and flexibility of the IVR tended to be transient and were linked to women's perceptions about their ability to insert or remove the ring. These concerns were mostly dispelled once a woman experienced actual use. Qualitative sub-study participants from the DR were more likely to express initial concerns about ring size than US participants. It is possible that, for some women, a lack of previous experience seeing and using an IVR, or other vaginal hygiene products gave rise to initial concerns. Overall, trial participants from both sites found the ring easy to insert and remove—particularly with some practice. These findings are line with those of a systematic review of vaginal ring acceptability for contraceptive or HIV indications from low- and middle-income countries. Across 68 studies, including both clinical trial and observational designs of different types of vaginal rings, most women rated their IVR experience as highly acceptable, and insertion and removal as easy (18, 22). Indeed, a recent literature review assessing barriers and enablers to women's uptake and use of vaginal contraception suggested that concerns about vaginal insertion as a disincentive to a product's demand are likely overestimated (23). Indeed, numerous studies suggest that intravaginal practices are common and are engaged in for cleaning purposes, sexual pleasure, and fertility control (24–26).

Relatedly, most women reported that neither they nor their partners were able to feel the ring once in place. In two cases, however, the placement of the ring was uncomfortable. Several Phase I trials of other rings also reported some instances when women could feel the ring or might experience some cramping (16, 27). In a Phase III trial of the dapivirine HIV prevention vaginal ring, participants reported experiencing heaviness and pelvic pain especially during initial months of the trial and equated this to improper placement of the ring. Some women also reported that partners could feel the ring, leading some to preemptively remove the ring prior to sex (28). While studies in women and providers in a range of geographies reported pre-insertion concerns about a partner's discomfort during sex, actual reported impacts on daily life and sexual experience were minimal (17, 18, 29, 30). Nevertheless, for some women an intravaginal ring will not be a viable option due to challenges with ring insertion, perceptions of anatomical incompatibility, and/or perceived or experienced discomfort by a sexual partner.

A second insight relates to the relative lack of impact of IVR use on the sexual experience compared to that of condom use. As noted in the previously published acceptability paper, most trial participants preferred a 3-month injectable (75% overall) to other prevention methods. More than half of participants overall, and 75% of women from the DR site reported the reason for this preference as not interrupting sex. Ease of use and discretion were also important reasons for this preference (13). In a qualitative study with adolescent and adult heterosexual men and women and men-who-have-sex-with-men in Cape Town, South Africa, acceptability of and preferences for new prevention technologies varied by population and were based on experiences with similar products and their fit with lifestyle and sexual contexts (31). Adolescent and adult women cited their inability to negotiate consistent condom use with partners and a prevailing threat of sexual assault when describing preferences for vaginal rings or an HIV vaccine. For women, vaginal rings and vaccines could be used discreetly and long-term, unlike oral PrEP, and were under a woman's control. Adult MSM preferred an HIV vaccine, whereas adult heterosexual men preferred an oral PrEP product that was more familiar. In contrast to other groups, heterosexual men expressed distrust of vaccines and injections in general (31).

A final insight was that women weighed certain trade-offs between duration of use and potential health effects they may perceive. Most women liked the idea of continuously using an IVR for several months at a time. Indeed, in our study, some qualitative sub-study participants envisioned using a vaginal ring like a woman might use an IUD. Others suggested that a longer duration of use would be acceptable if it were possible to periodically remove the ring to clean it. Women's desire to clean the ring may have been due to observing some ring discoloration from use during menses. As reported previously, most participants either experienced no change in menses or lighter bleeding and/or fewer days of bleeding during product use (12). It is unclear whether women who have used intravaginal products like the IUD, or whose menses are light, either naturally or due to the TFV/LNG ring, will have the same desire to periodically remove and clean their ring as expressed in this study. Furthermore, while this trial found continuous use of the 90-day TFV/LNG IVR to be safe, the safety of longer durations has not been studied (12). In an open-label trial with 120 Rwandan women randomized to NuvaRing, used intermittently or continuously for 3 months, vaginal yeast infections occurred in 22% of intermittent users and 27% of continuous users. Ten percent of continuous users reported lower abdominal pain vs. none in the intermittent arm (17). In a laboratory sub-study, investigators also evaluated biofilm build-up on 415 rings used during the Rwandan study. They found bacteria—both healthy lactobacilli and bacteria such as G. vaginalis and A. vaginae associated with vaginal microbiota dysbiosis—to be present on most rings. Additionally, the density and composition of ring biomass was associated with vaginal microbiota dysbiosis, although causality could not be determined (32, 33). Regarding the TFV/LNG ring tested in this study, three clinical trials have demonstrated that the ring does not adversely affect the vaginal microbiota (12, 34, 35). In anticipation of Phase 3 trials or post-trial introduction, developing clear messages about whether, when and/or how to clean the IVR and impact of cleaning methods on contraceptive/HIV effectiveness or vaginal health is essential.

## Conclusions

Our findings suggest overall high acceptability of the 90-day TFV/LNG IVR, but also point out that modifications to decrease the size and/or thickness of the ring and to possibly extend the duration of use could increase acceptability even more. Moreover, the mostly transient concerns about ring size and thickness expressed by women who are naïve to vaginal product use suggests the need for materials and/or communication strategies that can demystify the ring, how it is inserted and removed and where it sits. Finally, women's concerns about potential health effects with longer and more continuous use will require additional data and clear messages that inform women about the potential effects of ring removal and cleaning behaviors on effectiveness and vaginal health.

## Data Availability

Quantitative data will be made available without undue reservation. Access to deidentified qualitative data may be made available upon request.
